# A Highly Efficient Ag Nanoparticle-Immobilized Alginate-g-Polyacrylonitrile Hybrid Photocatalyst for the Degradation of Nitrophenols

**DOI:** 10.3390/polym12123049

**Published:** 2020-12-19

**Authors:** Imran Hasan, Charu Shekhar, Walaa Alharbi, Maymonah Abu Khanjer, Rais Ahmad Khan, Ali Alsalme

**Affiliations:** 1 Environmental Research Laboratory, Department of Chemistry, Chandigarh University, Gharuan, Mohali 140301, Punjab, India; imranhasan98@gmail.com (I.H.); charushekhar1998@gmail.com (C.S.); 2 Department of Chemistry, Faculty of Science, King Khalid University, P.O. Box-9004, Abha 62529, Saudi Arabia; Wal-harbe@kku.edu.sa; 3 Department of Chemistry, College of Science, King Saud University, Riyadh 11451, Saudi Arabia; moon-700p@hotmail.com (M.A.K.); krais@ksu.edu.sa (R.A.K.)

**Keywords:** Ag nanoparticle, polymer nanocomposite, surface plasmon resonance, photocatalysis, Pesticides

## Abstract

Herein, we report PAN-g-Alg@Ag-based nanocatalysts synthesis via in situ oxidative free-radical polymerization of acrylonitrile (AN) using Alg@Ag nanoparticles (Alg@Ag NPs). Various analytical techniques, including FTIR, XRD, SEM, TEM, UV–Vis, and DSC, were employed to determine bonding interactions and chemical characteristics of the nanocatalyst. The optimized response surface methodology coupled central composite design (RSM–CCD) reaction conditions were a 35-min irradiation time in a 70-mg L^−1^ 2,4-dinitrophenol (DNP) solution at pH of 4.68. Here, DNP degradation was 99.46% at a desirability of 1.00. The pseudo-first-order rate constant (*K*_1_) values were 0.047, 0.050, 0.054, 0.056, 0.059, and 0.064 min^−1^ with associated half-life (*t*_1/2_) values of 14.74, 13.86, 12.84, 12.38, 11.74, 10.82, and 10.04 min that corresponded to DNP concentrations of 10, 20, 30, 40, 50, 60, and 70 mg L^−1^, respectively, in the presence of PAN-g-Alg@Ag (0.03 g). The results indicate that the reaction followed the pseudo-first-order kinetic model with an R^2^ value of 0.99. The combined absorption properties of PAN and Alg@Ag NPs on copolymerization on the surface contributed more charge density to surface plasmon resonance (SPR) in a way to degrade more and more molecules of DNP together with preventing the recombination of electron and hole pairs within the photocatalytic process.

## 1. Introduction

The environmental and economic impact of pollutants and toxic effluent on water quality has reached crisis levels and is detrimental to human and marine life [1]. Of the various carcinogenic compounds commonly found in water sources, nitrophenols and their derivatives are among the most harmful pollutants discharged from various pharmaceutical, agricultural, manufacturing, and steel production industry processes [2,3]. 2,4-Dinitrophenol (DNP), which belongs to a class of water-soluble alkyl dinitrophenols that are volatile in steam [4], is a particularly lethal carcinogen linked to restricted cell growth even at concentrations of 1 ppm, causing skin allergies and hyperthermia [5]. According to USEPA, DNP is part of a list of “priority pollutants” with a permissible limit of 10 mg L^−1^; DNP is acidic at a pKa value of 4.03 [6]. Given its devastating effects to both human health and the environment, researchers are keen to find effective, economical methods to remove DNP from water sources. None of the numerous reported physical and chemical procedures for the sequestration of phenolic pollutants, including strategies based on precipitation, ion exchange, adsorption, and disinfection, have effectively removed DNP from water sources, as most require unacceptably long reaction times, are expensive, or are destructive [3,4,5]. Photodegradation, which is the oxidation and fragmentation of organic molecules into small non-toxic moieties under the aegis of solar light at UV or visible wavelengths, has been proposed as a sophisticated and efficient method for overcoming the shortcomings of the above-mentioned procedures [6]. Photocatalytic degradation of organic pollutants using solar irradiation is extremely economical compared to the other physical and chemical processes using artificial UV radiation, as it circumvents the need for electric power input [6,7]. Photocatalytic degradation is extremely useful for water remediation processes and the purification of wastewater because it results in the complete mineralization of the organic pollutants to generate harmless inorganic compounds [8,9].

Interdisciplinary research teams are currently working to characterize the chemical and physical properties of metal-based nanoparticles for photocatalytic applications. These nanoparticles have become renowned for their ability to remove organic pollutants from wastewater thanks to their large surface area, high adsorption capacity, weakened resistance to diffusion processes, and rapid reaction times due to the speed with which equilibration with guest molecules is achieved [10,11]. Various metal- and metal oxide-based nanocomposites, including ZnO [12], CuO [13], TiO_2_ [14], Fe_3_O_4_ [15], SnO_2_ [16], and Ag [17], have been utilized for the photocatalytic degradation of nitrophenols. In particular, silver (Ag) NPs are considered to be the most prevalent and effective noble metal nanoparticles due to their optical, electronic, thermal, medicinal, photocatalytic, and antibacterial properties [17]. Since Ag NPs fall in the metal category, the valence band (VB) and conduction band (CB) overlap [10]. Thus, in these cases, the photocatalytic properties of Ag NPs in visible light may emerge due to excitation of surface plasmon resonance (SPR), which is the manifestation of a resonance effect due to the interaction of conduction electrons of metal nanoparticles with incident photons. The interaction relies on the size and shape of the metal nanoparticles and on the nature and composition of the dispersion medium [18,19]. The high absorption property of Ag-based nanocomposites in the visible light region, together with preventing the recombination of electron and hole pairs within the photocatalytic process, has drawn enormous attention to the application of Ag NPs in the catalytic field [20]. Since these metal nanoparticles are also thermodynamically unstable due to agglomeration effects, a considerable effort was taken into consideration to functionalize Ag NPs using copolymer chains composed of alginate-co-polyacrylonitrile to facilitate more efficient charge transfer mechanisms, particularly for the degradation of DNP as part of a green chemistry approach to water purification. Alginate, an anionic biopolymer obtained from brown algae and bacteria, is comprised of L-guluronic acid (G) and D-mannuronic (M) residues that are directly linked by 1,4-glycosidic linkages [21]. According to the US Food and Drug Administration, alginate is known for its high gel porosity, biocompatibility, and stability in aqueous environments [22]. Poly(acrylonitrile) (PAN) is a synthetic polymer extensively used in hot gas filtration systems and for the production of outdoor awnings, sails for yachts, and fiber-reinforced concrete due to its supreme physical qualities, commercial availability, and environmental stability [23]. Studies have shown that PAN can be employed for photocatalytic degradation reactions at maximum irradiation power intensity over 2–6 h [24]. PAN is a readily available, inexpensive polymer that exhibits high thermal stability, making it the ideal supporting material for copolymerization with alginate to generate a matrix platform that can be embedded with silver nanoparticles. The experimental results suggest enhanced physical, chemical, and morphological properties of PAN-g-Alg@Ag compared to the individual constituents.

Response surface methodology (RSM) is recognized worldwide as the best statistical and mathematical tool for optimizing reaction parameters with good precession and high desirability values [25]. In addition to its use in various applications for the production of biological hydrogen and acetic acid during the acidogenesis of wastewater, bacterial growth, and enzyme synthesis, RSM is sequentially applied for azo dye decolorization and photodegradation processes via the implementation of biological and physicochemical techniques [26]. RSM reduces the need for replicating experiments during optimization studies [27]. Since the goal of the current study was to find the most efficient method for the photodegradation of DNP in wastewater under direct solar irradiation using synthesized PAN-g-Alg@Ag NC, the application of RSM in our study was particularly useful for optimizing the aforementioned reaction.

## 2. Materials and Methods

### 2.1. Chemicals

Sodium alginate (200 kDa, Na^+^ 10%) and acrylonitrile (AN, 99%) were acquired from Merck Co. Mumbai, India. 2,4-Dinitrophenol (DNP, C_6_H_4_N_2_O_5_ > 98%) and silver nitrate (AgNO_3,_ ACS reagent > 99.0%) were obtained from Sigma-Aldrich, Bangalore, India. All chemicals were used without any further modification, purification, or distillation. Deionized water (DI) was used for all experiments.

### 2.2. Synthesis of the PAN-g-Alg@Ag Nanocomposite Material

The synthesis of the respective materials was conducted using a previously reported protocol, but with some modifications [28,29]. Briefly, a solution containing 3.5% (*w*/*v*) alginate was added to a solution of AgNO_3_ (0.07 M) in DI water (100 mL), and the resulting mixture was stirred at 8000 rpm and 298 K for 5 h. The color of the mixture slowly became pale yellow, indicating the occurrence of nucleation of the Ag NPs supported by alginate side chains. An aliquot of the acrylonitrile monomer (15 mL) with a stoichiometric quantity of the N, N-methylene bisacrylamide (MBA) crosslinker (0.50 g) was added to the Alg@Ag suspension at 338 K. After 2 h of vigorous mixing, 20 mL of a 3% (*w*/*v*) ammonium persulfate solution were added dropwise to initiate the in-situ polymerization of AN. The post-polymerization reaction was stirred for an additional 5 h before quenching protocols were conducted using excess 0.15 M ferrous ammonium sulfate. The resulting milky white precipitate was collected via filtration and washed with an excess amount of DI water to remove any unreacted starting materials. Next, the precipitate was dried in a hot air oven at 323 K for 5 h before being stored for future photocatalytic experiments and characterization analysis.

### 2.3. Analytical Techniques Used for Product Characterization

The crystal structure and properties of the product polymer was determined using FTIR, XRD, transmission electron microscopy (TEM), energy dispersive X-ray (EDX), scanning electron microscopy (SEM), and differential scanning calorimetry (DSC). The type of bonding interactions and functional groups present in the nanocatalyst was determined via Fourier-transform infrared spectroscopy (FTIR) using a Perkin Elmer PE1600 spectrometer (USA). FTIR analysis was conducted in the 400–4000 cm^−1^ frequency range with the transmission mode. A Rigaku Ultima 1 V XRD diffractometer was employed to assess the crystalline structure of the nanocomposites. TEM was used to determine the particle size of the nanocomposites. Here, the elemental size and dispensation of the nanocomposite materials in the blended polymer matrix were determined using a JEM 2100 electron microscope (Japan). The surface morphological features, elemental identification, and both the chemical composition and homogeneity of PAN-g-Alg@Ag were determined via SEM combined with EDX (SEM; JEOL GSM 6510LV, Japan). DSC was conducted using a Mettler Toledo DSC822e calorimeter to determine the heat capacity and the enthalpies of crystallization and fusion; this allowed us to establish the thermal stability of the product nanocomposite. A Shimadzu UV-1900 UV–Vis double-beam spectrophotometer was utilized to analyze aliquots of the DNP samples after completion of the photocatalytic reaction.

### 2.4. Photocatalytic Experiments

The photocatalysis of the prepared samples was executed via the degradation of the DNP under direct solar irradiation at 25 °C. Briefly, 0.03 g of PAN-g-Alg@Ag was dispersed in 20 mL of the DNP solution (70 mg L^−1^) under direct solar irradiation for 35 min to facilitate equilibration on the photocatalyst’s surface for degradation process. The final concentration of DNP after completion of the degradation process was quantified using a UV–Vis spectrophotometer at maximum wavelength of 317 nm and was expressed by the following equation:

(1) $$\%\;\mathrm{Degradation}=\frac{C_{o}-C_{t}}{C_{o}}\times 100$$

where *C_o_* and *C_t_* are the concentrations of DNP before and after photodegradation.

## 3. Results and Discussion

### 3.1. Characterization of the Synthesized Nanocomposite

**Figure 1:**
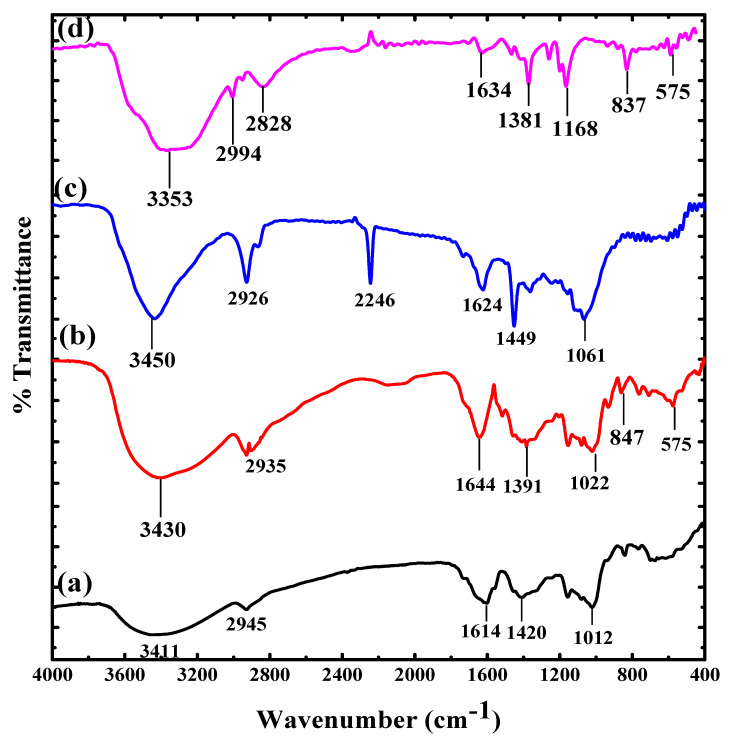
FTIR spectra of: (**a**) sodium alginate; (**b**) Alg@Ag NPs; (**c**) poly(acrylonitrile); and (**d**) PAN-g-Alg@Ag.

The FTIR spectra of PAN-g-Alg@Ag and its discrete elements are shown in Figure 1. The FTIR spectrum of sodium alginate (Figure 1a) shows peaks at 3411 cm^−1^ (–OH stretching), 2945 cm^−1^ (aliphatic –CH_2_ stretching), 1614–1420 cm^−1^ (symmetric and asymmetric –COO^−^ stretching), and 1012 cm^−1^ (pyranoid C–O–C stretching) [30]. The FTIR spectrum of the Alg@Ag NPs (Figure 1b) contains a peak at 575 cm^−1^ that corresponds to the Ag–O coordination bond, whereas the peak at 3430 cm^−1^ is due to –OH stretching and C=O bond stretching is evident in the peak at 1644 cm^−1^ [31]. The spectrum of PAN (Figure 1c) exhibits a wide sharp peak at 3450 cm^−1^ that is attributed to –OH groups due to humidity; the peak at 2246 cm^−1^ confirms the presence of nitrile groups (–C=N); peaks are noted at 1449–1624 cm^−1^ (–CH and –CH_2_ vibrations), 1061 cm^−1^ (–C–CN bond stretching), and 2926 cm^−1^ (stretching vibration of the –CH_2_ group of the PAN chain) [32]. The spectrum of PAN-g-Alg@Ag NC (Figure 1d) contains peaks at 3353 cm^−1^ (both –OH and –NH stretching) and 1634 cm^−1^ (–C=O bond). The peak at 575 cm^−1^ is due to the Ag–O coordination bond, while peaks within the range of 1168–1381 cm^−1^ are attributed to the symmetric and asymmetric –COO^−^ stretching of the alginate backbone. The reduced intensity of the peak associated with the nitrile functionality (2246 cm^−1^) is an indication of copolymerization between PAN and Alg rather than evidence of homo-polymerization of acrylonitrile [33,34].

**Figure 2:**
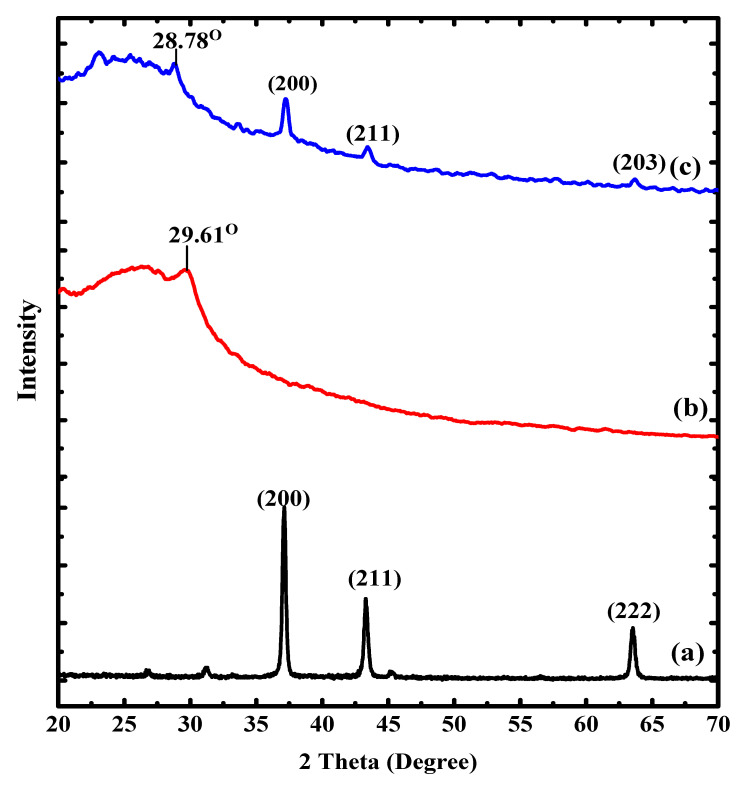
XRD spectra of: (**a**) Alg@Ag NPs; (**b**) poly(acrylonitrile); and (**c**) PAN-g-Alg@Ag.

Figure 2 displays the XRD spectra of PAN-g-Alg@Ag and its discrete components, providing information about the nanocomposite’s crystalline structure. The XRD pattern of the Alg@Ag NPs shows 2θ values of 37.11°, 43.32°, and 63.54°, corresponding to Miller indices of (200), (211), and (222), respectively, for the various planes of Alg@Ag NPs [35]. The XRD pattern shows that PAN is amorphous. The XRD spectrum of PAN-g-Alg@Ag contain all peaks associated with Alg@Ag NPs at 28.83° (from PAN), 37.24°, 43.44°, and 73.99° with the corresponding Miller indices of (200), (211), and (203), thereby indicating that the Alg@Ag NPs successfully copolymerized with the PAN chains.

Further information about the crystalline size cab was obtained using Scherer’s formula: [36]

(2) $$D=\frac{0.9\lambda }{\beta \cos\theta }$$

(3) $$\mathrm{Dislocation}\;\mathrm{Density}\;(\delta )=\frac{1}{D^{2}}$$

(4) $${\mathrm{Interlayer}\;\mathrm{Spacing}\;(d}_{200})=\frac{n\lambda }{2Sin\theta }$$

(5) $$\%\;\mathrm{Crystallinity}\;=\;\frac{\mathrm{Area}\;\mathrm{under}\;\mathrm{the}\;\mathrm{crystalline}\;\mathrm{peaks}}{\mathrm{Total}\;\mathrm{area}}\times 100$$

where *D* is the crystal’s size, *λ* is the wavelength used (i.e., 1.54 A°), *β* is the half-width of the most intense peak, and *θ* is the angle of diffraction. Equation (2) reveals that the average crystal size in both Alg@Ag NPs and PAN-g-Alg@Ag was around 20 and 8 nm, respectively. As noted in Figure 2, there is a great compression in the peak width and intensity due to the functionalization of Alg@Ag NPs with PAN. These interactions are vital for facilitating variations of the d-spacing and lattice distortions during aggregation, resulting in additional size compressions from 20 to 8 nm after functionalization. The XRD data show that the surface of Alg@Ag NPs has been successfully modified by the PAN polymer blend, resulting in well-dispersed, semi-crystalline solids with adequate functional density.

Table 1 shows the diffraction angle, FWHM, interlayer spacing, crystal size, dislocation density, and percent crystallinity of the pure, functionalized Alg@Ag NPs. Generally, PAN-g-Alg@Ag NC has smaller crystals (17.48 nm) and large dislocation density (3.27 × 10^16^) lines (m^−2^) at the (200) peak. The dislocation density (*δ*) is a measure of the number of defects in a crystal and is expressed in lines per meter square. The peak width and intensity of the product varies significantly depending on the extent of functionalization in the Alg@Ag NPs. The change in crystallinity from 52% for Alg@Ag NPs to 19% for the PAN-g-Alg@Ag nanocomposite is evidence of the copolymerization of Alg@Ag NPs by PAN chains.

**Figure 3:**
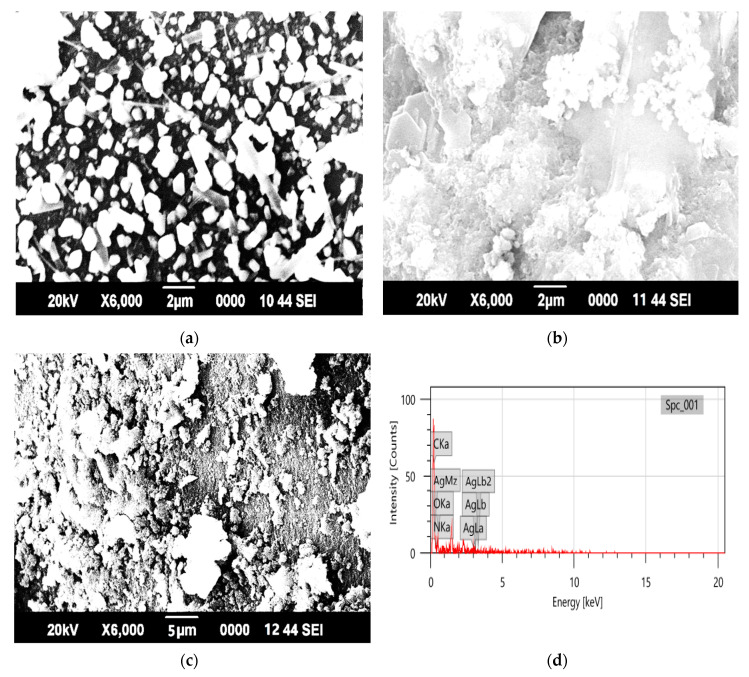
SEM images of: (**a**) sodium alginate; (**b**) poly(acrylonitrile); and (**c**) PAN-g-Alg@Ag. (**d**) The EDX spectrum of PAN-g-Alg@Ag.

The surface morphology of PAN-g-Alg@Ag was determined via SEM analysis, as illustrated in Figure 3a–d. Figure 3a represents the SEM image of alginate showing crystalline dots across the plating surface at 2 μm (×6000 magnification). The surface of PAN (Figure 3b) appears to be fuzzy and fibrous at 2 μm (×6000 magnification). After the copolymerization of PAN and Alg@Ag NPs, as shown in Figure 3c at 5 μm (×5000 magnification), PAN’s morphology appeared porous with embedded granules. The SEM images confirmed the synthesis of PAN-g-Alg@Ag. Figure 3d displays the EDX spectrum of PAN-g-Alg@Ag, providing information about the elemental identification, chemical composition, and chemical homogeneity of PAN-g-Alg@Ag. Here, the strong signals of the silver atoms are noted at 3.30 keV, which is consonant with the reported binding energy of Ag NPs in silver metallic nanocrystals [37]. The total elemental composition of PAN-g-Alg@Ag NC is given in Table 2 using the K-line spectrum.

**Figure 4:**
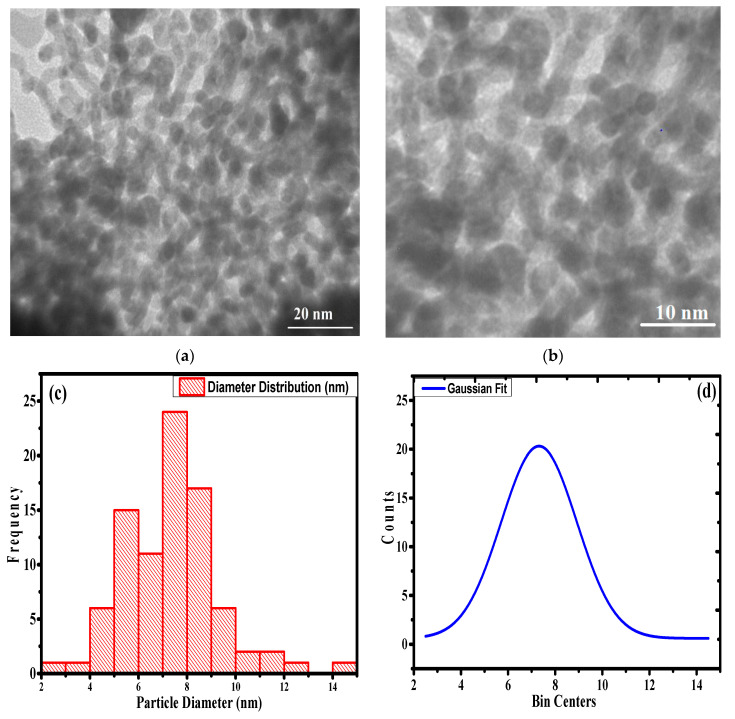
TEM images of the PAN-g-Alg@Ag nanocomposite.

The particle size and distribution inside the polymer’s stacked/unstacked matrix are established by conducting TEM measurements (Figure 4) at 20 and 10 nm magnification (Figure 4a,b, respectively). Here, we note that the minute globular point groups are limited and seem to be finely dispersed throughout the PAN-g-Alg copolymer matrix. The TEM image shows chemical homogeneity due to the presence of the PAN-g-Alg copolymer, which is ultimately responsible for stabilizing and reducing the Ag NPs. In addition, the average particle size of the Ag NPs in the PAN-g-Alg copolymer matrix is 8 nm, as determined via the Gaussian average particles size distribution curve given in Figure 4c,d. Elemental mapping analysis was utilized to analyze the atomic distribution share of individual atoms present in the PAN-g-Alg@Ag NC. The mapping images in Figure S1 reveal that the atomic share (%) of C, O, N, and Ag are 29.13, 37.64, 32.72 and 0.51 respectively.

**Figure 5:**
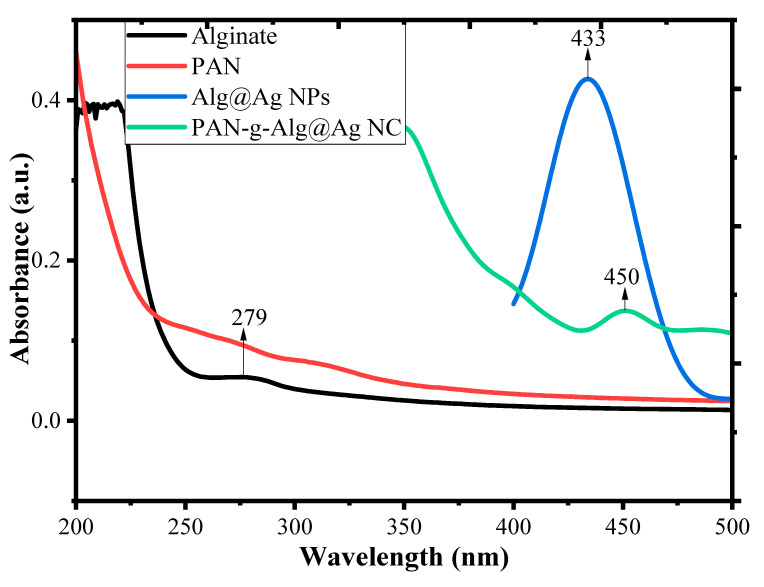
UV–Vis spectrum of alginate (black line), PAN (Red line), Alg@Ag NPs (blue line), and PAN-g-Alg@Ag NC (green line).

The optical absorption properties of the alginate, PAN, Alg@Ag NPs, and PAN-g-Alg@Ag NC were determined via UV–Vis spectroscopy in the wavelength range 200–500 nm (Figure 5). The UV–Vis spectra of alginate exhibit a low peak at 279 nm, while PAN shows negligible absorbance in the UV–Vis range. The spectra of Ag NPs synthesized through alginate sols show a remarkable absorbance peak at 433 nm, which is due to complete reduction of Ag^+^ ions into Ag NPs [38]. Further copolymerization of these Alg@Ag NPs with PAN chains resulted in a decrease in the magnitude of absorbance of Ag NPs, and a shift in the maxima was observed at 450 nm. Thus, a red shift in absorbance maxima from 433 to 450 nm with reduction in peak intensity suggested that the Alg@Ag NPs were successfully functionalized by PAN chains. The alignment of the absorbance maxima in the visible region suggested that the nanocomposite material may utilize the visible solar light more efficiently in photocatalytic reactions [10]. The photocatalytic activities of Ag NPs evolve out of excitation of SPR (surface plasmon resonance), which is simply a collection of oscillating surface electron gas propagating at the interface of metal and dielectric medium [20].

**Figure 6:**
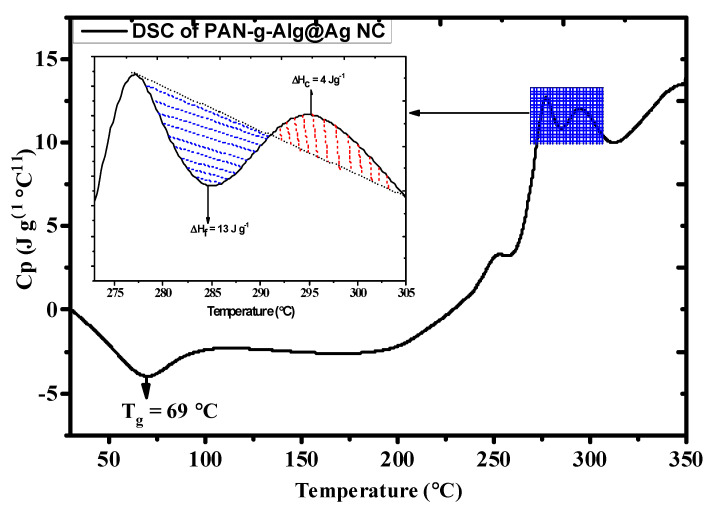
DSC spectrum of the PAN-g-Alg@Ag NCs.

DSC analysis is generally used to determine the thermodynamic stability of a compound by establishing various factors, including the glass transition temperature (*T_g_*) and the enthalpies of fusion or melting (Δ*H_F_*) and crystallization (Δ*H_C_*). The DSC profile of the nanocomposite material is given in Figure 6, and the inset contains an enlarged image of the selected area of analysis. The endothermic peak at 69 °C is attributed to the glass transition temperature (*T_g_*) of the polymer matrix functionalized with Ag NPs. The initial and final melting temperatures of the material were 277 and 291 °C, respectively, which was due to the occurrence of cyclization reactions of the nitrile group [34,38]. The heat capacity (*C_p_*) at the initial reaction temperature was 12.94 J g^−1^ °C^−1^. The enthalpy of fusion (Δ*H_F_*) was 13.08 J g^−1^, whereas the enthalpy of crystallization (Δ*H_C_*) was 4.53 J g^−1^.

### 3.2. The RSM-Coupled Approach and Statistical Exploration

Minitab17 software was utilized for conducting the experimental design that was subsequently executed via the RSM-coupled CCD to establish the synergistic or antagonistic effects of two or more variables on the response of the nanocomposite. The entire design mainly consisted of three parameters: irradiation time within the range of 10–35 min (A), a pH range of 1–5 (B), and the DNP concentration within the range of 20–70 mg L^−1^ (C). For the efficient degradation of DNP using PAN-g-Alg@Ag, the above-mentioned variables can be articulated using the quadratic regression equation:

(6) $$y=b_{0}+\sum_{i=1}^{n}b_{i}x_{i}+\sum_{i=1}^{n}b_{ii}x_{i}{}^{2}+\sum_{1\leq i<j}^{n}b_{i}{}_{j}x_{i}x_{j}+\varepsilon$$

where *x_i_* and *x_j_* represent the linear function that transforms the original actual values, *X_i_ − αC_i_*, *X_i_ − C_i_*, *X_i_*, *X_i_ + C_i_*, and *X_i_ + αC_i_*, to the coded values −α, −1, 0, +1, and α:

(7) $$x=\frac{\left(X-x_{i}\right)}{C_{i}}$$

As noted in Table S1, these three variables, namely the irradiation time (A) (10–35 min), DNP solution pH (B) (1–5), and DNP concentration (C) (20–70 mg L^−1^), were selected based on primarily batch-based experiments. Table S2 shows the variable ranges with the incorporated experimental and theoretical photodegradation responses. The correlation between the experimental and theoretical responses using the quadratic equation allowed us to effectively optimize the required reaction variables. Quadratic regression modeling was employed to determine the responses of the respective coded values for the three variables, which, in turn, was based on the experimental and theoretical outcomes:

%D = 108.20 − 0.43 A − 1.21 B − 0.20 C + 0.01 A^2^ − 0.01 B^2^ + 0.00 C^2^ − 0.01 A × B + 0.00 A × C + 0.04 B × C (8)

In this equation, the positive sign indicates the occurrence of synergism, whereas the negative sign is indicative of an antagonistic effect. Equation (8) shows that all the parameters (i.e., A, B, and C) exert antagonistic effects that influence the efficiency of photodegradation [39].

#### 3.2.1. Analysis of Variance

The statistical implication and interaction results of each term obtained from the quadratic model are manifested via the analysis of variance (ANOVA), as shown in Table 3. The respective coefficient terms and the significance of the regression model are evaluated by the *p* and F values using Fisher’s null hypothesis method. Here, increased applicability is associated with the quadratic relevance model, and each coefficient term is imposed by the small *p* and large F value. The large F and small *p* values confirm the model’s appropriateness, as evidenced by the RSM-coupled CCD [40]. The condition proposed by Fisher *p* > F < 0.05 can be seen in Table 3. Here, the reasonable *p* > F value of 0.0002 noted in the proposed quadratic regression model is statistically significant and relevant for the photocatalytic degradation of DNP on PAN-g-Alg@Ag NCs. Linear variable terms such as the irradiation time (A, *p* > F = 0.001), the DNP solution’s pH (B, *p* > F = 0.032), and the DNP concentration (C, *p* > F = 0.026) are statistically significant. When only the statistically significant terms in Equation (8) are taken into consideration, we obtain Equation (9):

%D = 108.20 − 0.43 A − 1.21 B − 0.20 C + 0.01A^2^ − 0.01 A × B + 0.04 B × C (9)

The amplitude of the design is estimated by associating the coefficients of R^2^ and R^2^_adj_. Here, the ANOVA values for R^2^ and R^2^_adj_ are 0.91 and 0.87, indicating that there is a correlation between the theoretical and experimental values of the photocatalyst’s response.

#### 3.2.2. Interpretation of the 3D Surface Data and Interaction Curves

**Figure 7:**
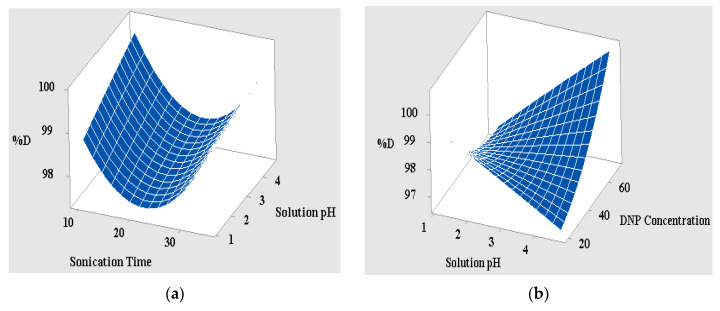

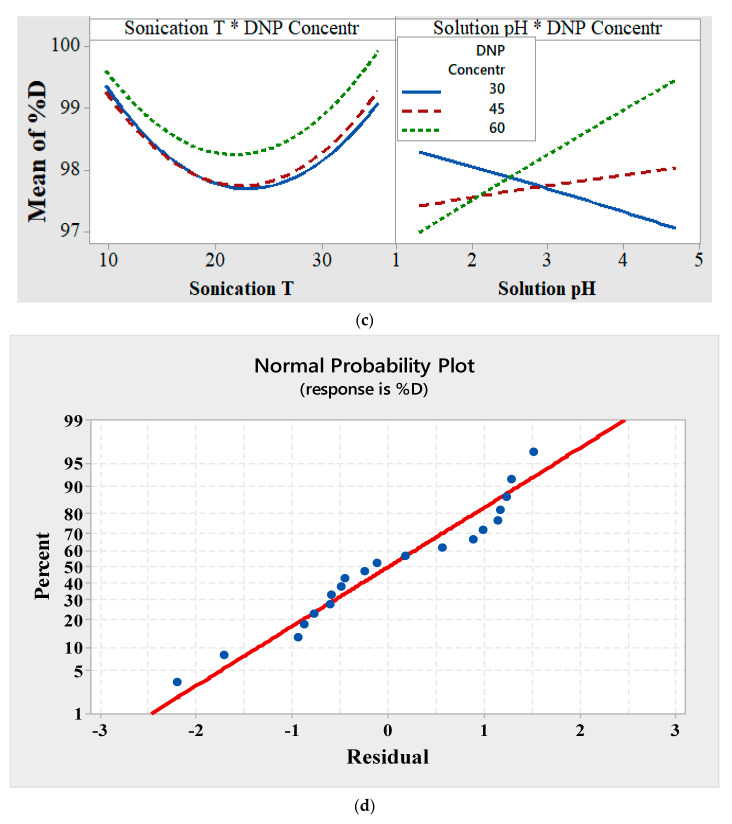
(**a**,**b**) 3D surface interactive plot of the irradiation time vs. solution pH and a plot of the solution pH vs. the DNP concentration; (**c**) interaction curves of all three reaction variables, namely irradiation time, solution pH, and DNP concentration; and (**d**) normal probability plot depicting the data points along the straight line.

The 3D surface designs are the graphic representations of the quadratic regression equation that describe the synchronous effect of two variables on the photodegradation reaction when the other variables are maintained. Figure 7a,b depicts the 3D surface interaction curve between the pH of the DNP solution and the irradiation time under direct solar irradiation when the other variable remains constant. Notably, both variables exhibit a gradual increase in the reaction time from 10 to 45 min, which is accompanied by the simultaneous increase in both the percent degradation from 95.22% to 99.80% and the pH of the solution from 1 to 5. It can be inferred from the results in Figure 7a that long radiation times and high pH values favor the degradation of DNP by PAN-g-Alg@Ag NC. The reason behind this behavior may be attributed to the large number of active pore sites on the surface that facilitate extensive host–guest interactions. As the reaction proceeds, increasingly more active sites become engaged in the photodegradation of DNP, resulting in a higher percent degradation under longer irradiation times [3]. Since pH 4.0–5.0 favors a high degradation capacity of 99.80%, this may be interpreted as the competitive interaction for particular active sites on the photocatalyst’s surface by both H^+^ and phenolate ions at pH < 4; this is reflected in the pKa value of 2,4-DNP (4.4) under strongly acidic conditions. An increase in the pH from 1 to 5 also reduces the number of available H^+^ ions, thereby allowing the maximum number of DNP molecules to interact with surface functional groups such as the cyano and hydroxyl moieties under direct solar irradiation. This, in turn, leads to a higher degradation capacity for the photocatalyst. At pH > 5.0, the presence of more negative ions generates repulsive forces between the negatively charged phenolate ions and the surface of the material, thereby decreasing the nanocomposite’s photocatalytic capacity [41]. Figure 7b shows the effects of increasing the DNP concentration from 20 to 70 mg L^−1^, which has the knock-on effect of increasing the photocatalyst’s degradation capacity from 95.22% to 99.80%. This improved catalytic efficiency facilitates the incorporation of increasingly more guest molecules through chemical interactions with the surface-active sites under direct solar irradiation. This, in turn, reinforces the principle that higher DNP concentrations favor improved percent degradation [42]. Figure 7c represents the interaction curve between the three operational variables, which follows the same trend observed above but with one notably different trait. Here, a rise in the DNP concentration simultaneously increases the nanocomposite’s photodegradation capacity and the irradiation time. Similarly, the plot consisting of the solution’s pH and the DNP concentration (i.e., 30, 45, and 60 mg L^−1^) shows a decrease in the nanocomposite’s photodegradation capacity at 30 mg L^−1^, and a converse increase in the catalyst’s photodegradation capacity at DNP concentrations of 45 and 60 mg L^−1^ with a simultaneous rise in the solution’s pH. This trend, observed at 30 mg L^−1^ of DNP, can be attributed to the availability of fewer DNP molecules for the active sites on the photocatalyst’s surface, resulting in a reduction in the catalyst’s photodegradation capacity. Figure 7d portrays the normal probability plot obtained for the design validation system; it can also be argued that the points dispersed across the straight line represent no reaction exchange and manifest a distinctive appropriation curve [26]. The figure highlights the transference of points across the straight line with the approximation of R^2^ and R^2^_adj_ as 0.91 and 0.87, respectively, thereby confirming the statistical validity of the model. The results obtained from the CCD model, which shows the three optimized variables after validation of the regression equation with a 95% confidence interval, are a 35-min irradiation time (A), a pH value of 4.7 for the DNP solution (B), and a DNP concentration of 70 mg L^−1^ (C). The photocatalytic experiments using PAN-g-Alg@Ag NC in the presence of these optimized conditions gives a maximum of 99.56%, which is close to the predicted value of 99.80% with a desirability value of D = 1.00.

### 3.3. Kinetics of Photodegradation

**Figure 8:**
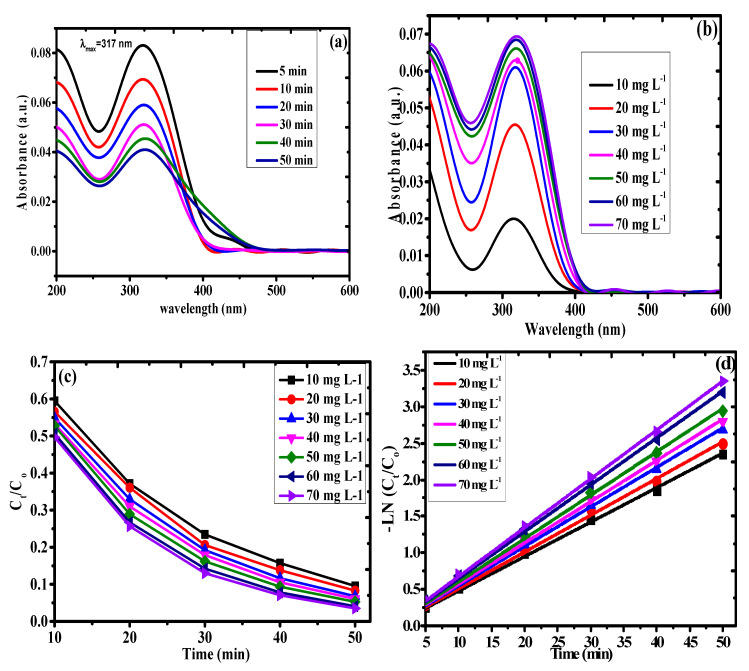
(**a**) UV–Vis action curve for irradiation time; (**b**) UV–Vis action curve for variable concentration of DNP; (**c**) Ce/Co vs. irradiation curve; and (**d**) pseudo-first order kinetic ci = curve for variable concentration of DNP.

The extent of the DNP photodegradation using the PAN-g-Alg@Ag NCs was investigated via UV–Vis spectroscopy of DNP (70 mg L^−1^) at the maximum absorbance of 317 nm (Figure 8a). The absorption spectra of the degraded 2,4-DNP aqueous solution during variable direct solar irradiation times in the presence of the photocatalyst is shown in Figure 8a. The absorbance peaks of DNP continuously decrease from an initial value of 0.083 at 5 min to 0.069 at 10 min, 0.058 at 20 min, 0.051 at 30 min, 0.045 at 40 min, and 0.041 at 50 min, indicating a rise in the total photodegradation capacity from 92.90% at 5 min to 96.49% at 50 min.

Figure 8b shows the absorbance spectra obtained using various concentrations of DNP ranging from 10 to 70 mg L^−1^ as a function of the irradiation time between 5 and 50 min. In the figure, we note that successive increases in the irradiation time from 5 to 50 min and the concentration of DNP from 10 to 70 mg L^−1^ results in a gradual decrease in the absorbance peak of DNP. The total photodegradation capacity under the optimized irradiation time of 50 min changes from 87.86% at 10 mg L^−1^ to 94.68% at 70 mg L^−1^. Incremental changes in the irradiation time during photocatalysis facilitate faster reactions with a degradation potency of 94% within a 50-min period.

Estimating the rate determining step in the kinetic experiments is vital for determining the reaction pathway in which the substrate adheres to the catalyst. The experimental evidence obtained from the pseudo-first-order kinetic model studies can be used to calculate the half-life periods and rate constants throughout the reaction [43]. Mathematically, this can be expressed as:

(10) $$-\ln\left(\frac{C_{t}}{C_{o}}\right)=kt$$

(11) $$t_{1/2}=\frac{0.693}{k}$$

where *C_o_* represents the original concentration of DNP (mg L^−1^), *C_t_* is the remnant concentration of DNP at time *t*, *k* is the pseudo-first-order rate constant (min^−1^), and *t*_1/2_ (min) represents the half-life of the reaction. Here, the concentration of DNP, i.e., 10, 20, 30, 40, 50, 60, and 70 mg L^−1^, is analyzed using the PAN-g-Alg@Ag NCs under the optimized reaction conditions. The results of the photodegradation studies are shown in Figure 8c as a plot of *C_t_*/*C_o_* against the irradiation time. A comparable plot between –Ln(*C_t_*/*C_o_*) and the irradiation time (min) is seen in Figure 8d. Here, the slope of the straight line represents the first-order rate constant (*K*_1_) as 0.047, 0.050, 0.054, 0.056, 0.059, and 0.064 min^−1^, with half-life (*t*_1\2_) values of 14.74, 13.86, 12.84, 12.38, 11.74, 10.82, and 10.04 min, respectively, which correspond to 10, 20, 30, 40, 50, 60, and 70 mg L^−1^ of DNP with 0.03 g of PAN-g-Alg@Ag. The upgraded pseudo-first-order kinetic model, with an R^2^ value of 0.99, is shown in Table 4. Thus, the outcome of these optimized experiments on the photocatalysis of DNP using PAN-g-Alg@Ag NCs is that incremental increases in the DNP concentration with respect to the irradiation time leads to gradual increases in the rate of photodegradation up to 35 min. Beyond this point, the higher light absorbance of the DNP molecule in aqueous solution facilitates effective photodegradation on the surface of PAN-g-Alg@Ag. There is a noted light masking effect in the aqueous medium due to the increased number of DNP molecules that impede the absorption of light by the photocatalyst’s surface [44]. The incremental increase in the magnitude of this masking effect generates the reactive radical (H•O) or electron–hole $\left(h_{vb}^{+}\right)$ induced by excitation of SPR that subsequently degrades the DNP molecule. The rate determining step in this heterogeneous catalysis normally entails the adsorption of DNP onto the surface of the PAN-g-Alg@Ag NC and its degradation through the reactive (H•O)/$\left(h_{vb}^{+}\right)$ species [4,44]. We deduce that the interference of the pathlength of the quantum of light during its interaction with the photocatalyst occurs as the DNP concentration increases, thereby retarding the rate of the degradation reaction. For an aliquot of DNP (20 mg L^−1^), the surface area of PAN-g-Alg@Ag required for efficient photodegradation can be easily calculated. As we move toward higher DNP concentrations, i.e., 40, 50, 60, and 70 mg L^−1^, the photodegradation process suffers from the limitations caused by a secondary layer of DNP molecules, which expands the duration of the irradiation parameter to facilitate the aggregation of the predominantly DNP molecular layer on the surface of the photocatalyst.

### 3.4. Photocatalytic Activity of PAN-g-Alg@Ag NC and Its Individual Entities

**Figure 9:**
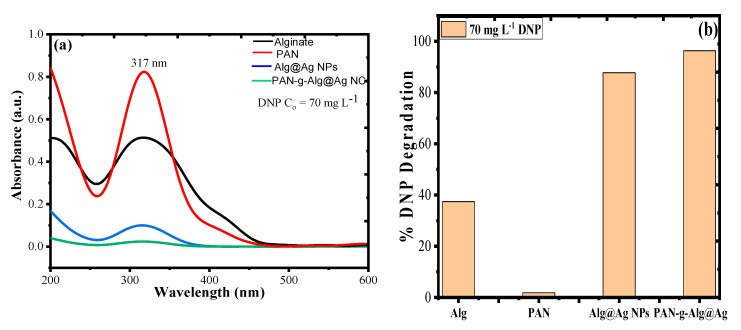
(**a**) UV–Vis plot for the photocatalytic activity of synthesized PAN-g-Alg@Ag NC and its individual constituents towards DNP; and (**b**) the corresponding percent degradation bar graph.

Figure 9a,b represents the UV–Vis plot for the photocatalytic activity of synthesized PAN-g-Alg@Ag NC and its individual constituents towards DNP and corresponding percent degradation rate under solar light irradiation and optimized reaction conditions. It can be seen from the results that the synthesized material has maximum photocatalytic efficiency (96.32%) as compared to its individual constituents of alginate (37.42%), PAN (1.84%), and Alg@Ag NPs (87.73%). The enhanced photocatalytic activity of PAN-g-Alg@Ag NC may be due to combined absorption properties of PAN and Alg@Ag NPs, which on copolymerization on surface contributed more charge density to SPR in a way to degrade more and more molecules of DNP [19].

### 3.5. Photocatalytic Degradation of DNP in Dark and Sunlight

**Figure 10:**
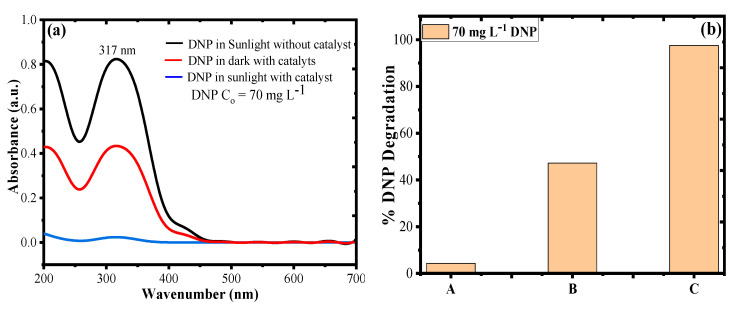
(**a**) UV–Vis plot; and (**b**) degradation rate of DNP in direct solar irradiation without catalyst (photolysis, A), dark with catalyst (B), and direct solar irradiation with catalyst (C) for an aliquot of 20 mL of 70 mg L^−1^ DNP under optimized reaction conditions.

Figure 10a represents the UV–Vis plot and degradation rate of DNP in direct solar irradiation without catalyst (photolysis), dark with catalyst, and direct solar irradiation with catalyst. Figure 10b shows that the rate of DNP degradation without catalyst under direct solar irradiation (photolysis, A) was found to be 4.29%, with catalyst in dark (B) was 47.23%, and with catalyst in direct solar irradiation (C) was 97.56% for an aliquot of 20 mL of 70 mg L^−1^ DNP under optimized reaction conditions. Since some part of DNP absorbs in visible region, which resulted in negligible degradation of DNP in direct solar irradiation without catalyst [45], a removal of 47.23% of DNP in the dark by catalyst was due to adsorption process. Since the catalyst is rich in surface functional OH/CN groups, which can bond the DNP to surface of the catalyst by some kind of adsorption process at optimal pH conditions, the combination of DNP with catalyst under direct solar irradiation at optimized reaction conditions resulted in 97.56% DNP degradation, which was due to photocatalytic mineralization of DNP by excited SPR of Ag NPs functionalized by PAN-g-Alg polymer blend.

### 3.6. Effect of Electrolyte Concentration on DNP Degradation

Figure S2 represent the UV–Vis plot for the effect of electrolyte concentration (NaCl) on the photocatalytic degradation of DNP under direct solar irradiation and optimized reaction conditions. The experiments were performed by taking 1–4 mM NaCl concentration with combination of DNP and catalyst. It can be seen that presence of electrolyte NaCl has very negligible antagonistic effect on the degradation of DNP as the results were 92.88% degradation at 1 mM NaCl, 93.01% degradation at 2 mM NaCl, 93.25% degradation at 3 mM NaCl, 94.64% degradation at 1 mM NaCl, and 96.21% degradation in absence of NaCl. Thus, with increase in salt concentration from 1–4 mM, the DNP degradation efficiency reduces from 96.21% to 92.88%.

### 3.7. The Mechanism of Photodegradation

Based on the data obtained through photocatalytic experiments for DNP degradation by PAN-g-Alg@Ag NC through excitation of SPR, a hypothesis was proposed to explain the type of mechanism involved in the photocatalytic degradation of DNP [46].

(12) $$\mathrm{PAN}\text{-}g\text{-}\mathrm{Alg}@\mathrm{Ag}+\mathrm{Solar}\;\mathrm{irradiation}\to {\mathrm{PAN}\text{-}g\text{-}\mathrm{Alg}@\mathrm{Ag}(h}_{\mathrm{VB}}^{+}{+e}_{\mathrm{CB}}^{-})$$

(13) $$\mathrm{DNP}\;+\;\mathrm{Solar}\;\mathrm{irradiation}\to {\mathrm{DNP}}^{*}$$

(14) $${\mathrm{DNP}}^{*}+\;\mathrm{PAN}\text{-}g\text{-}\mathrm{Alg}@\mathrm{Ag}\to {\mathrm{DNP}}^{•+}+{\mathrm{PAN}\text{-}g\text{-}\mathrm{Alg}@\mathrm{Ag}(e}_{\mathrm{CB}}^{-})$$

(15) $$O_{2}+{\;\mathrm{PAN}\text{-}g\text{-}\mathrm{Alg}@\mathrm{Ag}(e}_{\mathrm{CB}}^{-})\to \mathrm{PAN}\text{-}g\text{-}\mathrm{Alg}@\mathrm{Ag}+O_{2}^{•-}$$

(16) $$O_{2}^{•-}+{\;H}^{+}\to {\mathrm{HO}}_{2}^{•}$$

(17) $$2{\mathrm{HO}}_{2}^{•}\to O_{2}+H_{2}O_{2}$$

(18) $$H_{2}O_{2}+{\;O}_{2}^{•-}\to {\mathrm{HO}}^{-}+{\mathrm{HO}}^{•}+O_{2}$$

(19) $$H_{2}O_{2}+{\;e}^{-}\to {\mathrm{HO}}^{-}+{\mathrm{HO}}^{•}$$

(20) $$H_{2}{O\;+\;\mathrm{PAN}\text{-}g\text{-}\mathrm{Alg}@\mathrm{Ag}(h}_{\mathrm{VB}}^{+})\to \mathrm{PAN}\text{-}g\text{-}\mathrm{Alg}@\mathrm{Ag}+{\mathrm{HO}}^{•}+H^{+}$$

(21) $${\mathrm{HO}}^{-}+{\mathrm{PAN}\text{-}g\text{-}\mathrm{Alg}@\mathrm{Ag}(h}_{\mathrm{VB}}^{+})\to \mathrm{PAN}\text{-}g\text{-}\mathrm{Alg}@\mathrm{Ag}+{\mathrm{HO}}^{•}$$

(22) $$\mathrm{DNP}+{\mathrm{PAN}\text{-}g\text{-}\mathrm{Alg}@\mathrm{Ag}(h}_{\mathrm{VB}}^{+}{,\;O}_{2}^{•-},{\mathrm{HO}}^{•})\to \to \to \mathrm{CO}2\;+\;\mathrm{NO}2\;+\;H2O\;+\;\mathrm{other}\;\mathrm{byproducts}$$

**Figure 11:**
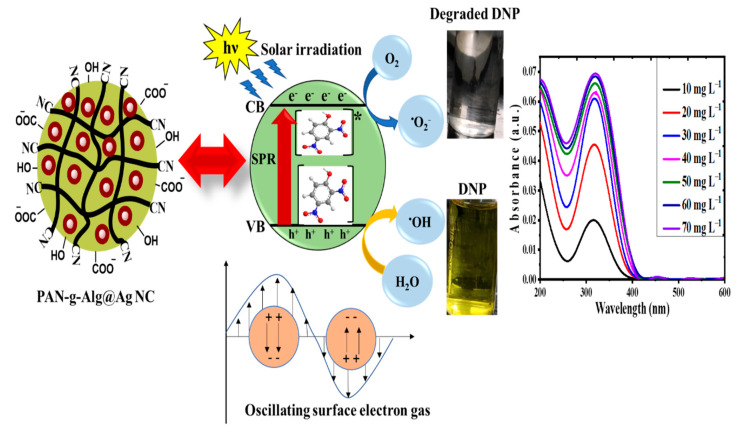
Photodegradation mechanism of DNP by PAN-g-Alg@Ag under direct solar irradiation.

The absorption of solar radiation on the surface of the PAN-g-Alg@Ag NC leads to excitation of SPR, which resulted in generation of charge carriers and transportation of electron and hole pairs (e^–^_CB_/h^+^_VB_). From the UV–Vis absorption experiments, it was found that both PAN-g-Alg@Ag and DNP can absorb solar irradiation efficiently. When a combination of PAN-g-Alg@Ag + DNP was placed under direct solar irradiation of energy hν, PAN-g-Alg@Ag absorbed the photons and generated (h^+^_VB_ + e^–^_CB_) pair due to their high SPR effect (Equation (1)) [47]. The adsorbed DNP molecule on catalyst surface absorbed the solar irradiation and donated its photogenerated electron to conduction band (CB) of PAN-g-Alg@Ag (Equations (2) and (3)). The surface adsorbed oxygen (O_2_) molecules in contact with these photogenerated electrons transformed into superoxide radical anions (O_2_^•−^) and hydrogen peroxide radicals (HOO^•^) (Equations (4)–(7)) [48,49]. As a result of SPR excitation, the photogenerated holes (h^+^_VB_) reacted with surface adsorbed water molecules (H_2_O) or hydroxyl anion (OH^−^) to transform them into hydroxyl radicals (HO^•^) [49]. These photogenerated active radical species then attacked the DNP molecule to mineralize it to nontoxic entities. The possible mechanism for degradation of DNP under direct solar irradiation is given in Figure 11. In support of this mechanism, total organic carbon (TOC) and chemical oxygen demand (COD) experiments were performed, as given in Figure S3.

### 3.8. Regeneration and Reusability of PAN-g-Alg@Ag NC and Alg@Ag NPs

One of the important problems associated with advance oxidative technology is the safe disposal of the spent catalyst. This hurdle was later overcome by researchers through the regeneration process, which involves the recovery of the spent catalyst for reuse, thus reducing the cost of the experiment. In the present study, regeneration and reusability experiments were performed for both PAN-g-Alg@Ag NC and Alg@Ag NPs by solvent washing method in which 0.03 g of catalysts after DNP degradation process was mixed with 20 mL of 0.1 M HNO_3_ solution and placed under sonication equipped with magnetic stirrer. The catalysts were collected by filtration and washed by distilled water until the effluent pH becomes neutral and dried in hot air oven for 2 h at 60 °C. The recovered catalysts were again utilized for the photodegradation of DNP at the optimized conditions under direct solar irradiation, and the results for photocatalytic efficiency of both PAN-g-Alg@Ag NC and Alg@Ag NPs are given in Figure S4. In the first cycle, the DNP degradation rates for the two catalysts were 97.23% and 88.75%, respectively. Further unitizing the catalysts for second, third, fourth, and fifth cycles using the same procedure resulted in percent DNP degradations of 94.64% and 85.32%, 91.88% and 81.98%, 89.11% and 75.39%, and 84.67% and 62.64%, respectively. In five cycles, the decrement of only 12.56% in photocatalytic efficiency of PAN-g-Alg@Ag NC as compared to Alg@Ag NPs (26.11%) suggested that the synthesized PAN-g-Alg@Ag NC is highly efficient and chemically more stable than Alg@Ag NPs for treatment of water contaminated by DNP due to functionalization of polymer blend of PAN.

### 3.9. Comparison with Literature

The advantage of using PAN-g-Alg@Ag NC was explored by comparing the degradation efficiency of the material towards DNP with previously reported studies. The results given in Table 5 suggest that the present study with 99.46% DNP degradation in just 35 min under visible solar irradiation was highly efficient as compared to the other reported photocatalysts.

## 4. Conclusions

Herein, we report the synthesis of PAN-g-Alg@Ag nanocomposites via the in situ oxidative free-radical polymerization of acrylonitrile using environmentally friendly Alg@Ag nanoparticles. Various analytical techniques, including FTIR and XRD, were used for characterization studies. We note the reduced intensity of the nitrile functional groups at 2246 cm^−1^ in the FTIR spectrum as an indication of copolymerization between PAN and Alg, with a corresponding peak at 575 cm^−1^ for the Ag–O coordination bond. The change in the crystal size of the Alg@Ag NPs from 20 to 8 nm in the PAN-g-Alg@Ag NC, as verified via XRD, is evidence of the modification of the surface of Alg@Ag NPs through polymerization with PAN, which results in well-dispersed, semi-crystalline solids with adequate functional density. RSM-coupled optimization of the reaction parameters indicate that a photocatalytic capacity of 99.46% with a desirability of 1.00 can be achieved when the irradiation time is 35 min, the pH of the DNP solution is 4.68, and the DNP concentration is 70 mg L^−1^. Additionally, the reaction is governed by pseudo-first-order kinetics, with the rapid generation of the OH radical at high DNP concentrations due to the masking effect exerted on the adsorption of DNP molecules on the PAN-g-Alg@Ag NC surface. The combined absorption properties of PAN and Alg@Ag NPs on copolymerization on surface contributed more charge density to SPR in a way to degrade more and more molecules of DNP together with preventing the recombination of electron and hole pairs within the photocatalytic process. In five cycles, the decrement of only 12.56% in photocatalytic efficiency of PAN-g-Alg@Ag NC as compared to Alg@Ag NPs (26.11%) suggested that the synthesized PAN-g-Alg@Ag NC is highly efficient and chemically more stable than Alg@Ag NPs for treatment of water contaminated by DNP due to functionalization of polymer blend of PAN. The future implications of this research will allow researchers to develop more efficient materials and reaction routes for the photodegradation of carcinogenic compounds.

## Figures and Tables

**Table 1:** XRD parameters of Ag NPs associated with the Alg and PAN-g-Alg copolymer matrix.

Component	2θ	FWHM (β_hkl_)	Interlayer Spacing (d_200_) (A°)	Size of Crystal (nm) at (200)	Dislocation Density (*δ*) × 10^15^ Lines (m^−2^)	% Crystallinity (%)
Alg@Ag NPs	37.11	0.33	0.26	25.39	1.55	52
PAN-g-Alg@Ag NC	37.24	0.48	0.24	17.48	3.27	19

**Table 2:** The EDX spectroscopic data of the PAN-g-Alg@Ag NC.

Element	Mass %	Atom %	Binding Energy (KeV)
C	24.03	29.13	0.26–0.29
N	36.21	37.64	0.44–0.46
O	35.95	32.72	0.52–0.54
Ag	3.80	0.51	3.00–3.20

**Table 3:** Analysis of variance (ANOVA) for the photodegradation of DNP on PAN-g-Alg@Ag NC.

Source	D_F_	A_dj_ SS	A_dj_ MS	F Value	*p* > F Value
Model	9	8.48	0.94	0.44	0.00
A	1	0.00	0.00	0.99	0.00
B	1	0.45	0.45	0.21	0.03
C	1	1.03	1.02	0.48	0.03
A^2^	1	3.99	3.99	1.86	0.00
B^2^	1	0.00	0.00	0.00	0.98
C^2^	1	0.75	0.75	0.35	0.57
A×B	1	0.03	0.03	0.02	0.01
A×C	1	0.06	0.06	0.03	0.87
B×C	1	2.39	2.39	1.11	0.03
Error	10	21.51	2.15		

A (irradiation time), B (DNP solution pH), C (DNP concentration), A × B (irradiation time × DNP solution pH), A × C (irradiation time× DNP concentration), B × C (DNP solution pH × DNP concentration).

**Table 4:** Pseudo-first-order kinetic model for the photodegradation of DNP by PAN-g-Alg@Ag NC with the associated error analysis.

S.N.	Concentration (mg L^−1^)	Rate Constant (*K*) (min^−1^)	Half-Life (*t*_1/2_) (min)	R^2^	SSE (×10^−4^)
1	10	0.05	14.74	0.99	5.13
2	20	0.05	13.86	0.99	6.79
3	30	0.05	12.84	0.99	4.72
4	40	0.06	12.38	0.99	5.54
5	50	0.06	11.74	0.99	5.41
6	60	0.06	10.82	0.99	3.81
7	70	0.07	10.04	0.99	3.44

**Table 5:** Comparison of degradation efficiency of PAN-g-Alg@Ag NC with previously reported studies.

Material/Photocatalyst	Irradiation Time (min)	pH	Kinetics	% Degradation of DNP	Reference
g-C_3_N_4_/AgI/ZnO/CQD	120 min	4	Langmuir–Hinshelwood	98%	[12]
Ag_2_CO_3_/PSGCN	120 min	4	Pseudo-first order	98%	[17]
CMIP-coated TiO_2_	240 min	5	Pseudo-first order	76%	[43]
ZnFe_2_O_4_	15 min	3	Pseudo-first order	82%	[4]
[Ag_4_(NO_3_)_4_(dpppda)]n	300 min	4	Pseudo-zero order	93%	[50]
ζ-Bi_2_O_3_/Bi_2_MoO_6_	100 min	7	Pseudo-first order	86%	[51]
ZnFe_2_O_4_-ZrO_2_	60 min	4	Pseudo-first order	90%	[8]
molecularly imprinted TiO_2_	240 min	4	Pseudo-first order	74%	[14]
Ag/CuO/TiO_2_	80 min	7	Pseudo-first order	99%	[13]
PAN–g–Alg@Ag	35 min	4.68	Pseudo-first order	99.46%	Present Study

## Supplementary Materials

The following are available online at https://www.mdpi.com/2073-4360/12/12/3049/s1, Table S1: Range of the variable parameters; Table S2: Design table including the number of experiments with respect to the obtained experimental and predicted data; Figure S1: Elemental Mapping for C, O, N, and Ag elements constituting PAN-g-Alg@Ag NC; Figure S2: (a) UV–Vis plot; and (b) degradation rate of DNP vs. concentration of NaCl to observe the effect of electrolyte concentration (NaCl) on the photocatalytic degradation of DNP under direct solar irradiation and optimized reaction conditions; Figure S3: Mineralization of DNP during the photodegradation with PAN-g-Alg@Ag NC using direct solar irradiation: (**a**) total organic carbon (TOC); and (**b**) chemical oxygen demand (COD); Figure S4: Regeneration and reusability graphs for PAN-g-Alg@Ag NC and Alg@Ag NPs towards DNP degradation.

## Abbreviations

Alg	Alginate
PAN	Poly(acrylonitrile)
Alg@Ag NPs	Alginate functionalized Ag nanoparticles
DNP	2, 4-Dinitrophenol
FTIR	Fourier-transform Infrared
SEM	Scanning Electron Microscope
TEM	Transmission Electron Microscope
XRD	X-ray Diffraction
EDX	Energy-dispersive X-ray spectroscopy
DSC	Differential Scanning Calorimetry
APS	Ammonium Persulfate
FAS	Ferrous Ammonium Sulfate
