# Supplementary material for: A Highly Efficient Ag Nanoparticle-Immobilized Alginate-g-Polyacrylonitrile Hybrid Photocatalyst for the Degradation of Nitrophenols
Source: Polymers (Basel). 2020 Dec 19;12(12):3049. doi: 10.3390/polym12123049 (PMC7766039; doi:10.3390/polym12123049)
Supplement: Supplementary file 1 [file polymers-12-03049-s001.pdf]

Supporting Information

A Highly Efficient Ag Nanoparticle-Immobilized Alginate-g-Polyacrylonitrile Hybrid Photocatalyst for the Degradation of Nitrophenols

Imran Hasan, [1] Charu Shekhar, [1] Walaa Alharbi,[2] Maymonah Abu Khanjer,[3] Rais Ahmad Khan[3] and Ali Alsalme,[3,*]

[1] Environmental Research Laboratory, Department of Chemistry, Chandigarh University, Gharuan, Mohali, Punjab, 140301, India; imranhasan98@gmail.com (I.H.), charushekhar1998@gmail.com (C.S.);

[2] Department of Chemistry, Faculty of Science, King Khalid University, P.O. Box-9004, Abha-62529 Saudi Arabia; Wal-harbe@kku.edu.sa (W.A.);

[3] Department of Chemistry, College of Science, King Saud University, Riyadh-11451, Saudi Arabia, moon-700p@hotmail.com (M.A.K.), krais@ksu.edu.sa (R.A.K.);

* Correspondence aalsalme@ksu.edu.sa (A.A.)

Table 1. Range of the variable parameters.

Variable	Unit	-2	Low factorial (-1)	Center (0)	High factorial (+1)	+2
Radiation Time	(min)	10.00	15.00	22.50	30.00	35.11
pH		1.32	2.00	3.00	4.00	4.68
CV Concentration	(mg L^{-1})	20.00	30.00	45.00	60.00	70.22

Table 2. Design table consisting the number of experiments with respect of obtained experimental and predicted data.

S.N.	Sonication Time	Solution pH	DNP Concentration	%Degradation	Predicted %Degradation
1	22.50	3.00	45.00	98.92	97.75
2	22.50	3.00	45.00	96.87	97.75
3	15.00	4.00	60.00	98.98	99.47
4	22.50	3.00	45.00	99.04	97.75
5	35.11	3.00	45.00	98.64	99.25
6	22.50	3.00	45.00	97.16	97.75
7	22.50	3.00	70.23	97.91	98.85
8	10.00	3.00	45.00	98.78	99.23
9	15.00	4.00	30.00	97.76	97.99
10	22.50	3.00	20.00	97.82	97.93
11	22.50	3.00	45.00	98.64	97.75
12	15.00	2.00	30.00	99.17	98.60
13	22.50	3.00	45.00	96.04	97.75
14	30.00	4.00	30.00	96.94	97.71
15	30.00	2.00	60.00	99.18	98.19
16	15.00	2.00	60.00	99.40	97.88
17	22.50	1.32	45.00	95.22	97.42
18	22.50	4.68	45.00	99.17	98.03
19	30.00	2.00	30.00	99.80	98.57
20	30.00	4.00	60.00	99.70	99.52

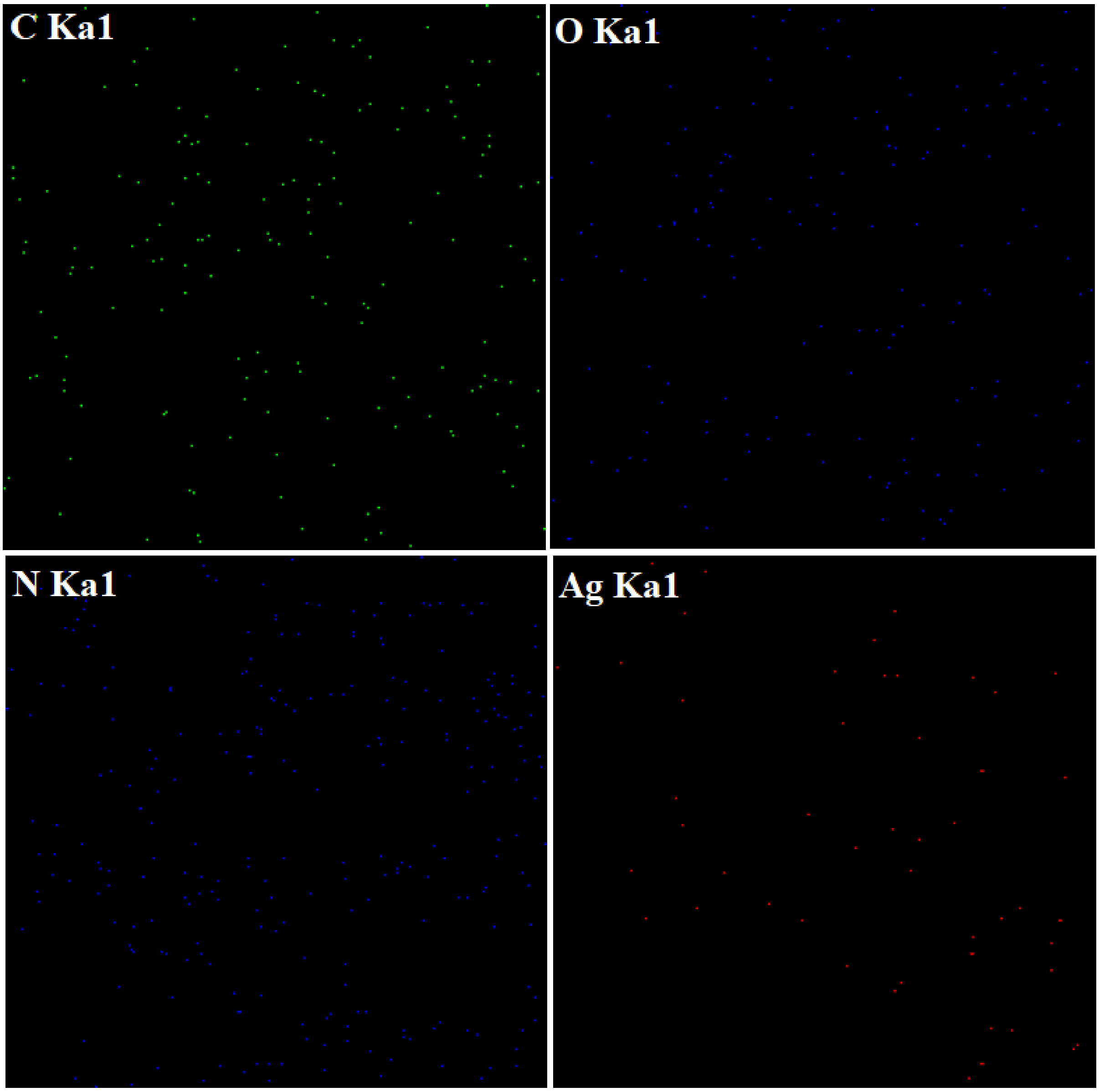

Figure 1. Elemental Mapping for C, O, N and Ag elements constituting PAN-g-Alg@Ag NC.

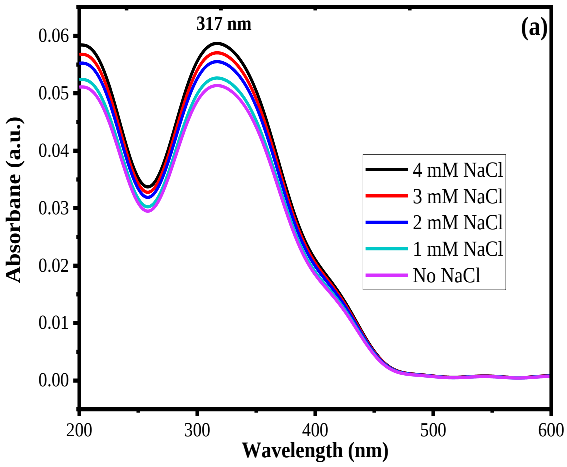

(**a**)

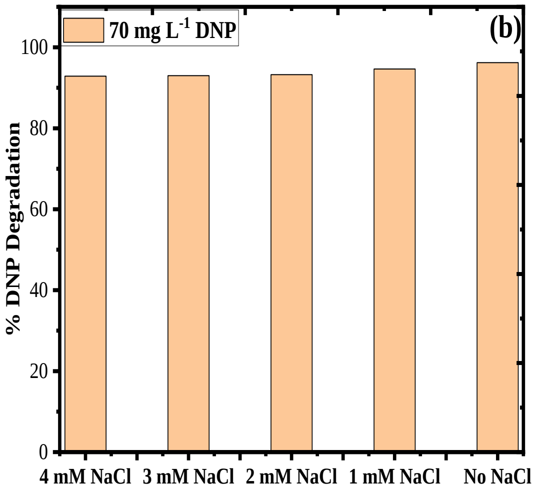

(**b**)

Figure 2. (a) UV-Vis plot (b) Degradation rate of DNP vs concentration of NaCl to observe the effect of electrolyte concentration (NaCl) on the photocatalytic degradation of DNP under direct solar irradiation and optimized reaction conditions.

Mineralization of DNP

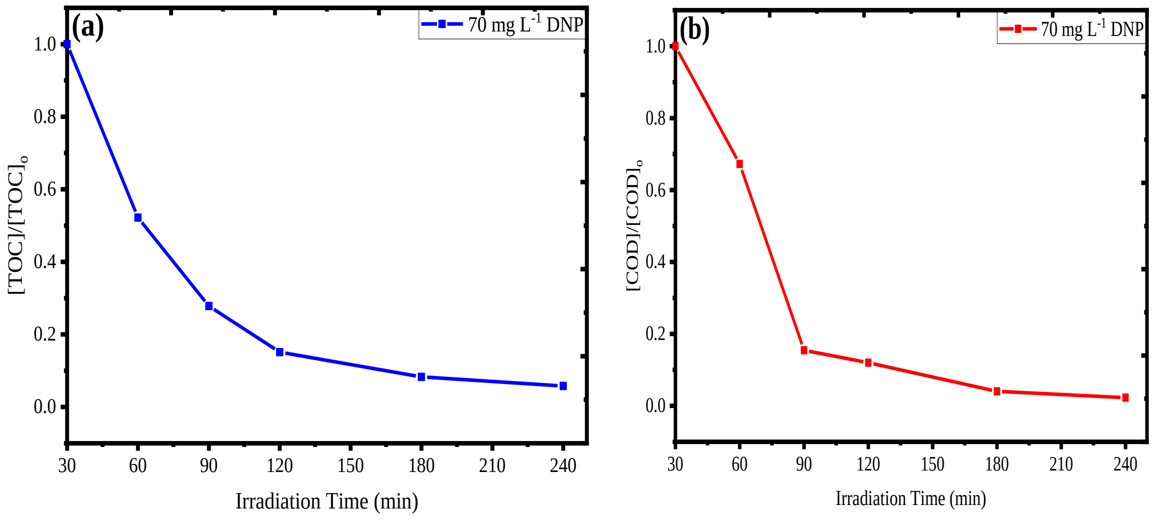

(a) **(b)**

Figure 3. Mineralization of DNP during the photodegradation with PAN-g-Alg@Ag NC using direct solar irradiation (**a**) total organic carbon (TOC) and (**b**) chemical oxygen demand (COD).

The conversion of DNP into nontoxic entities like CO_2, H_2O etc was observed by measuring the total organic carbon (TOC) and chemical oxygen demand (COD) studies. Figure S3a represents the TOC plot for the degradation of DNP under direct solar irradiation in which it was inferred that after 120 min of irradiation time the TOC value rapidly decreased up to 85.21%. Further increase in irradiation time up to 240 min results in a total of 94.36% reduction in TOC value for photodegradation of DNP using PAN-g-Alg@Ag NC. Figure S3b represents the variation in chemical oxygen demand (COD) with respect to the photodegradation of DNP. The purpose of the test was to analyse the amount of oxygen needed for the conversion of DNP into CO_2 and H_2O. It can be inferred from the Figure S3b that with increase in solar irradiation time from 30 min to 240 min the COD value decreased with an extent of 98% indicating the complete mineralization of DNP by PAN-g-Alg@Ag NC.

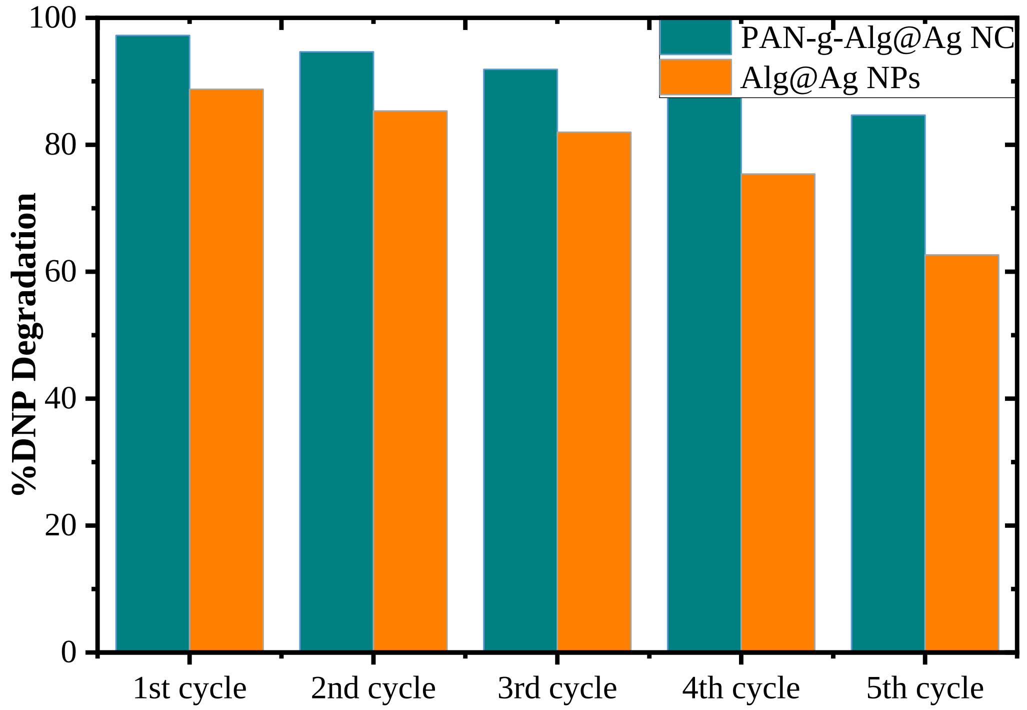

Figure 4. Regeneration and reusability graphs for PAN-g-Alg@Ag NC and Alg@Ag NPs towards DNP degradation.
